# New Contact Sensorization Smart System for IoT e-Health Applications Based on IBC IEEE 802.15.6 Communications

**DOI:** 10.3390/s20247097

**Published:** 2020-12-11

**Authors:** David Hernández, Rafael Ors, Juan V. Capella, Alberto Bonastre, José C. Campelo

**Affiliations:** Instituto ITACA de Tecnologías de la Información y Comunicaciones, Universitat Politècnica de València, 46022 València, Spain; dhernandez@itaca.upv.es (D.H.); rors@itaca.upv.es (R.O.); jcapella@itaca.upv.es (J.V.C.); bonastre@itaca.upv.es (A.B.)

**Keywords:** intrabody communications, interbody communications, IEEE 802.15.6

## Abstract

This paper proposes and demonstrates the capabilities of a new sensorization system that monitors skin contact between two persons. Based on the intrabody communication standard (802.15.6), the new system allows for interbody communication, through the transmission of messages between different persons through the skin when they are touching. The system not only detects if there has been contact between two persons but, as a novelty, is also able to identify the elements that have been in contact. This sensor will be applied to analyze and monitor good follow-up of hand hygiene practice in health care, following the “World Health Organization Guidelines on Hand Hygiene in Health Care”. This guide proposes specific recommendations to improve hygiene practices and reduce the transmission of pathogenic microorganisms between patients and health-care workers (HCW). The transmission of nosocomial infections due to improper hand hygiene could be reduced with the aid of a monitoring system that would prevent HCWs from violating the protocol. The cutting-edge sensor proposed in this paper is a crucial innovation for the development of this automated hand hygiene monitoring system (AHHMS).

## 1. Introduction

Nowadays, the society is immersed in a complete transformation due to the great impact that new technologies are causing. Terms like “Internet of Things” (IoT), “Ambient Intelligence” (Ami) or “Smart Systems” (such as SmartCities, SmartHomes, SmartHospitals, etc.) are more and more commonly employed and are becoming part of our daily lives. However, part of the success in the implementation of these applications is conditioned by the development of new and more sophisticated sensor systems that provide the necessary information for their correct operation.

The health area is not alien to this transformation, which is evident by the increasing quality of health services thanks to the emergence of new applications of Information and Communications Technologies (ICT) in this field, among other factors. Our research line is currently focused on a project in the field of e-health, which centers on the monitoring of hand hygiene protocols.

The World Health Organization (WHO), aware of the impact that good hygienic practices have on the health of the global population, has promoted several actions in this line. One of these initiatives has published a report, entitled “WHO Guidelines on Hand Hygiene in Health Care” [1], to provide health-care workers (HCWs), hospital administrators and health authorities with a thorough review of evidence on hand hygiene in health care and propose specific recommendations to improve good practices and reduce transmission of pathogenic microorganisms to patients and HCWs. This guide emphasizes the importance of preventing the health care-associated infection (HCAI), by giving priority to the promotion of hand hygiene best practices in health care.

Additionally, the WHO identifies five moments for hand hygiene in practice, being necessary to carry out not only awareness campaigns, but also the analysis and monitoring of the good follow-up of these practices in different countries. 

Studies about the hand hygiene practices show a degree of compliance between 60% and 70% in the better cases. Nevertheless, in some sectors the compliance falls as low as 8% [2,3].

Concretely, to carry out the monitoring of proper hand hygiene in hospitals and increase the hygiene protocols compliance, the WHO identifies three techniques:

1. Monitoring hand hygiene by direct observation. The hand hygiene compliance is measured by means of validated observation. In hand hygiene compliance, this is considered the preferred standard.
2. Indirect monitoring of hand hygiene performance. Health professionals have estimated the number of hygiene actions based on products consumption such as alcohol-based hand rub, paper towels or liquid soap.
3. Automated Hand Hygiene Monitoring Systems (AHHMS). Electronical devices may detect and report the use of sinks and hand rub dispensers. Although it can be known when an HCW use those electronical devices, an in-depth research must be done to know when HCWs are in physical contact with patients and thus need to disinfect after this interaction.

Although direct observation is considered the gold standard when measuring compliance with hand hygiene protocols, this observation method suffers from the Hawthorne effect [4], which refers to the tendency of the observed subject to modify its behavior when observed. 

The estimation based on the consumption of products related to hand hygiene, such as soap or alcohol-based hand rub, does not allow for the evaluation of individual behaviors, nor the adoption of preventive measures.

There are many AHHMS described in the literature, and some of them even became commercial devices. Between indirect monitoring and AHHMS, some systems automatically observe the use of the dispensers, computing the use of consumables in real time. Other systems [5], in addition, identify the HCW that performs the hand hygiene event (i.e., cleaning), and its entry and exit from patient rooms [6,7,8]. Others rely on localization techniques [9] to locate HCWs, detecting their proximity to dispensers (hand hygiene event) and to beds occupied by patients (hand hygiene opportunity—the moment at which the HCW changes from a safe state to an unsafe state where a hand hygiene disinfection is required before approaching another patient) [10,11,12]. Many of these AHHMS employ active RFID devices and beacons [13] to implement the location algorithm. Finally, there are AHHMS [14] in the literature that use monitoring cameras [15,16] or subjective vision cameras [17] to monitor HHP compliance, although a human visualization of the recordings is required to detect both hygiene events and opportunities.

Whereas each hand hygiene event performed by the HCW may be easily detected with most of these technologies, it is not possible to automatically ensure when a hand hygiene opportunity occurs. If a HCW gets close to a patient, but there is no contact between them, there is no reason for a later disinfection. The HCWs are aware of this, and therefore forcing them to use the dispensers after this interaction causes them boredom and discouragement.

The weak point of these AHHMS can be found in the lack of automatic detection of hand hygiene opportunities.

In this paper we present a novel approach towards a sensor system that is able to detect when a person inside a hospital (either a health worker or a patient) has come into contact with other people, and even more, is able to identify the order in which the sequence of contacts has been done. Depending on this sequence, there may be risks for a person’s own health or that of other people. In this way, after each contact, the involved person is considered to require a hand cleaning procedure (unsafe state) before his possible contact with other people. Therefore, it is possible to warn him when he approaches other persons before the contact, making him to be aware of this state and thus preventing the possible risks. Obviously, after a correct cleaning procedure, such as using a hydroalcoholic solution dispenser, he is considered to become safe again.

As can be seen, the success of this application depends on three different sensors: (i) the use of dispensers, and (ii) proximity and (iii) contact with other people. Sensors that indicate whether a dispenser has been used, as already indicated above, are already in the commercialization phase. Sensors that indicate the proximity of a person to certain objects are also common, even by means of a location system or measuring the Received Signal Strength Indicator (RSSI) of a transmitted signal, ultrasound echo, etc. 

The last required sensors must be able to detect the contact between people and even identify the involved individuals. 

Indeed, when facing this hospital sensorization project, the first task was a search to find on the market any commercialized sensor (or set of sensors) capable of detecting the contact between two persons and identifying them. Unfortunately, no results were found, not even under development.

As the result was negative, we proceeded to review the available scientific literature looking for proposals of this type of sensors. Although there are some different proposals for sensors that are capable of detecting contact, these systems are not able to identify the object or person that has been touched. 

As none of these techniques offered the desired characteristics, we decided to investigate the development of a new sensorization system that met the aforementioned specifications. Our starting point were several studies found in the scientific literature about the transmission of signals through the skin, which led us to study the IEEE 802.15.6 standard [18] as the basis of the new sensorization system. The use of a standard, in addition to obtaining universality, ensures that the proposed sensor system can be used in parallel with other compatible applications. On the other hand, the use of these systems opens the door to obtaining new sensorization environments that offer great possibilities for future applications. So, this is the main goal of this paper: it shows a proof of concept through the development and validation of a prototype to enlighten the capabilities of using interbody communication through the skin to identify contact between two persons. This information, when merged with the one obtained from dispensers and proximity sensors, will allow for the identification of risk situations (infection possibilities).

Finally, the design of the proposed sensorization system, must also take into account the following characteristics:

- Easy installation. These sensors should be installed and used by people from the hospital facility.
- Identification of unlimited (or a very large) number of different people.
- Reliability. False positives and undetected contacts must be kept below 1%.
- Touch detection on any part of patient’s body.
- Safety. These sensors cannot influence other hospital systems, especially the most critical ones.

The results of this investigation are presented in this work. This paper is organized as follows: after the introduction, Section 2 shows the related work. Section 3 describes our proposal. The system description is explained in Section 4. After that, the experimentation and results are detailed in Section 5 and Section 6, respectively. Finally Section 7 shows the conclusions of this work.

## 2. Related Work

### 2.1. Wireless Personal Area Sensor Networks and Body Area Networks

The progressive miniaturization of computer systems and the advances in transducers for the observation of parameters related to health, have allowed for the emergence of small devices for monitoring the activity and well-being of people. These sensor devices usually use low-power wireless networks, the so-called WPANs (Wireless Personal Area Networks), to make their measurements available to devices with greater calculation and communication capabilities. Bluetooth [19] and ZigBee [20] may be considered as examples of these technologies.

The difference between a WBAN (Wireless Body Area Network) and a WPAN is relevant. The WPAN covers the space around the person, covering distances of up to a hundred meters. On the other hand, a WBAN allow the communication of devices deployed in or over a human body, covering distances around two meters. Examples of a WPAN network are Bluetooth (IEEE 802.15.1) or ZigBee (IEEE 802.15.4). Otherwise, a WBAN network may use technologies such as UWB (Ultra-Wide Band), NB (Narrow Band), or HBC (Human Body Channel). HBC is standardized in IEEE 802.15.6 [18].

It is commonly accepted that Zimmerman [21] published the earliest research of WBAN in 1996. From this milestone, many approaches to this issue can be found in scientific publications. Wireless transmission is one of the most popular in WPAN and WBAN, with many proposals based on standards (Bluetooth [22], ZigBee [23], Ultra-wideband [24], Narrowband [25], and even WiFi [26]) and non-standard wireless communications [27,28,29].

Privacy issues [30,31], ISM band saturation, power loss, and interferences with medical equipment and patient health [32,33,34] are some of the reasons that recommend the use of the so-called Intra Body Communications (IBC) [35]. Previous proposals have studied the propagation of RF signals [36,37], capacitive [38,39,40] and galvanic coupling [41,42] and even ultrasonic [43,44] emissions through the human tissues. This way, Intra-body Communication (IBC) is becoming the most appropriate alternative for devices that require low power consumption, information security, frequent reuse, and resistance to interference. In addition, an IBC device requires a small form factor, allowing portability and not obstructing the mobility of the user. There are basically two transmission mechanisms for IBC: capacitive coupling and galvanic coupling. In the first method, the electric signal is controlled by an electric potential, while in the second method it is controlled by a current flow. 

The IEEE 802.15.6 standard [18], published in 2012, was intended to stablish a common communication environment for WBANs. From the three physical layers available at the standard (Narrow Band, Ultra-Wide Band, and Human Body Channel, HBC) the last has been hardly studied, and a very few implementation efforts can be found [45]. This promising method of transmission will allow the development of devices to adhere to the human body or even to implant under the skin. 

The most significant requirements for this new technology are:

- Safe for the user
- Respond to the requirements in terms of bandwidth (not excessively high) and error rate (the lower the better), but allowing activity peaks for emergencies
- Preserve the confidentiality of information as much as possible.
- Energy efficient, thus allowing to increase the technology’s useful life.
- Light and small size, that is, not very intrusive.

### 2.2. IEEE 802.15.6 Standard for Intra-Body Communication

The IEEE 802.15.6 standard [18] was designed for WBANs. Both ISM (industrial scientific medical) band and other frequencies approved by national medical and/or regulatory authorities are used for this purpose. 

The main characteristics of this standard are:

- Short-range, covering the human body range.
- Low power, making possible self-powered devices.
- Highly reliable wireless communication.
- Typical data rates up to 10 Mbps.
- Meets the medical and relevant communication regulations.
- Support for Body Area Network (BAN) applications.

**Figure 1:**
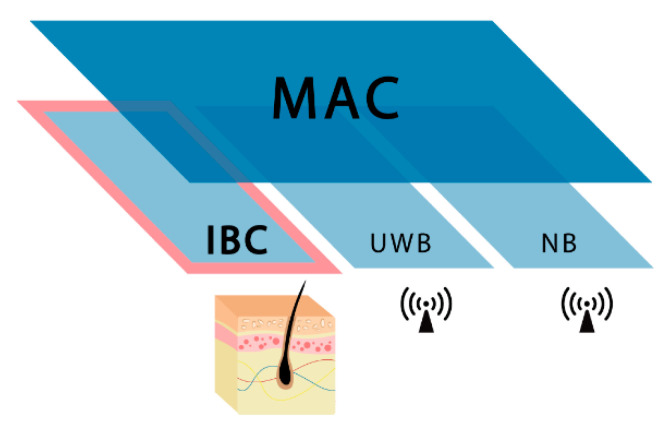
IEEE 802.15.6 MAC and PHY layers.

Three different physical layers (PHY), using different frequency ranges, are defined, namely Narrow Band (NB), Ultra-Wide Band (UWB), and a physical layer specifically designed for Intrabody communications (IBC, HBC, or BCC). They are shown in Figure 1. The first two operate through radio waves, using the frequency bands determined by the standard and their objective is the communication around the body. Nevertheless, IBC is a designed for communications through human tissues, essentially the skin. For the research presented in this paper, only THE IBC physical layer is considered. 

### 2.3. 802.15.6. IBC PHY Layer

The PHY layer of HBC does not use RF communication and thus antennas are not required, different from NB and UWB. The communication technology used, Electric Field Communication (EFC), requires the use of electrodes attached to the human body, in order to use the skin as a communication channel. EFC is based in the fact that positive terminals of both transmitter and receiver are connected to the human body and negative terminals are opened to keep the system ground-free. This way, the environment provides the signal return path.

The frequency selective digital transmission (FSDT) scheme is adopted for the IBC transmission through the human body. This technique is based in spreading the data with frequency selective Walsh code prior to transmission directly in digital form. Walsh codification provides a unique fundamental frequency for each code sequence. 

Regarding the frequency band of operation for PHY HBC, the standard reflects that a compatible device must be able to support data transmissions and receptions in the 21 MHz band [18] and operates with a bandwidth of 5.25 MHz [18].

Four data rates are proposed in the standard: depending on the receiver minimum sensitivity a data rate from 1641 to 13,125 kbps is achievable. This is because the HBC transmitter uses the frequency selective digital transmission (FSDT) scheme, where Walsh code are used to spread the information bits. The data rate of this system is limited to 1.3125 Mbps, due to the limited set of spreading codes available for use under the transmission mask of IEEE802.15.6.

**Figure 2:**
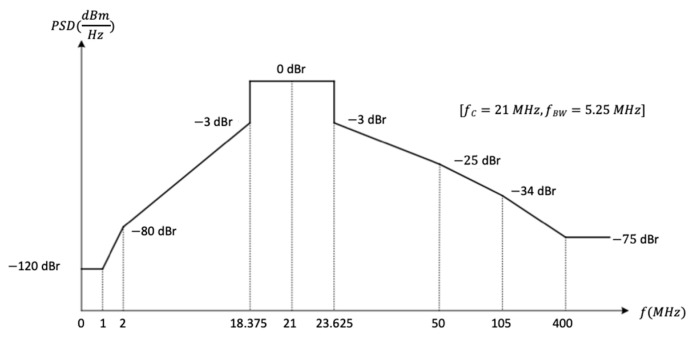
802.15.6 Transmit spectral mask.

Furthermore, it is important to highlight that a transmit spectral mask shall be used to remove harmonics and possible interferences in other bands, especially with 400 MHz medical band. The transmit power spectrum will be less than 0 dBr (dB relative to the maximum spectral density of the signal) within f_BW_. In case that f_C_ is 21 MHz, f_BW_ is 5.25 MHz, where f_C_ is channel central frequency and f_BW_ is channel bandwidth. The required transmit spectral mask is shown in Figure 2 for 21 MHz channel central frequency.

## 3. Proposal and Implementation

**Figure 3:**
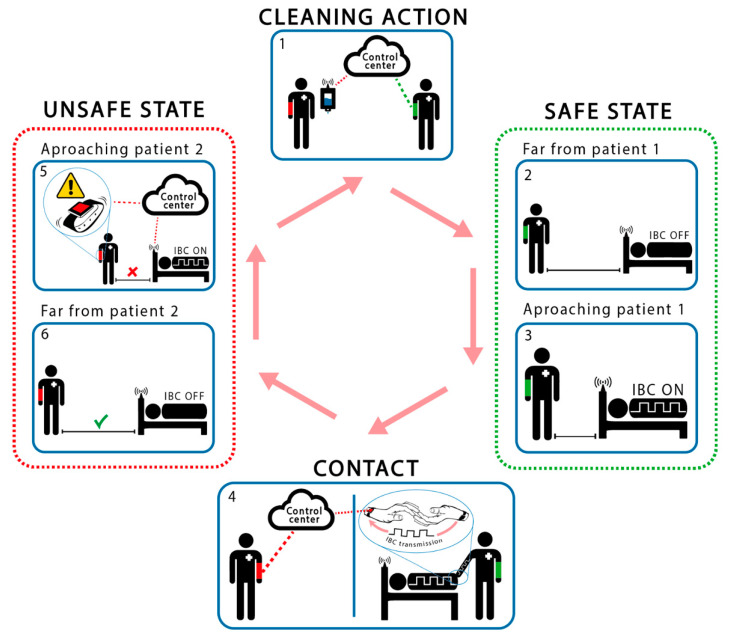
Operation of the proposed AHHMS.

Figure 3 shows the operation of the AHHMS where the proposed sensing system will be integrated. Although this paper focus on the new contact sensorization system, this section outlines the behavior of the whole system to clarify its operation and the relationships between subsystems. 

As mentioned before, the application monitors and controls the accomplishment of the hands cleaning protocols. As it can be seen in Figure 3, several sensorization subsystems are necessary to achieve the desired application. A Smart Hospital Control Center, based on Cloud technology, is in charge of storing in a data base the actions related to the hand hygiene protocol. It also controls its accomplishment and offers a friendly user interface. All required data is provided by different sensorization subsystems. The first subsystem is related to the hydroalcoholic solution dispenser devices, and informs about its use. The second subsystem consists of a proximity sensorization system that detects when a health worker gets close to a patient or a dispenser. And finally, the proposed contact sensorization system, that is based on the HCWD (Health Care Worker Device) and the HCPD (Health Care Patient Device), that detect contact between these agents and identify them. As it has been said before, while the first and second subsystem do not present a research challenge, the last one does. So, this last system is the main object of this research paper.

From all data obtained from all subsystems, a specific software in the Control Center will obtain the measures of interest: the identification of non-compliance with protocol restrictions, and therefore the risk of spreading nosocomial infections due to improper hygiene, and many other related parameters that medical staff would require. 

Both health care workers and patients will carry their corresponding HCWD or HCPD, each one of them with a unique identifier: Object_ID. These Object_IDs can be transmitted from a HCWD to a HCPD when a skin-contact exists between persons (interbody communication). This is the main difference with respect to proximity systems and the main contribution of this paper. Additionally, these user devices will have connectivity with other protocols such as WiFi and Sub-Ghz to allow for communication with the server and for proximity detection as explained below. This way, the contact between health care workers and patients will be registered in the Control Center.

As part of the hospital facilities, each bed and hydroalcoholic dispenser (Figure 3) will be equipped with a proximity subsystem. It is used to prevent the potential HCAI, and to avoid continuous transmission of the user devices through the skin. Our first prototype uses a Sub-GHz wireless sensor network to detect when the HCW approaches them. The range of detection is programmable based on the Received Signal Strength Indicator (RSSI) level. 

When an HCW begins its work, it is considered to be in an “unsafe” state (potentially contagious), so its first action must be to use a hydroalcoholic dispenser. This action is detected by the sensor of the dispenser and registered into the control system. At this time, the HCW passes its status to “safe”. Every time that this HCW approximates to a patient, the proximity sensors activate the HCPD contact transmitter. If the HCWD receives the Object_ID from the HCPD, a contact between HCW and patient is detected. In this case, the HCWD registers this action, including both Object_IDs, into the control system by means of a WLAN. At this time, the HCW changes its status to “unsafe” and only this patient may be touched again without risks. After that, the HCW must perform a cleaning action before getting close to another patient. To do this, the HCW must approach and use a hydroalcoholic dispenser. This action changes its status to “safe”, and the HCW is allowed to approach another patient. If the HCW gets near of other patient before this cleaning action, the proximity sensor detects this approach in “unsafe” mode and the Control Center is informed of this protocol violation. At the same time, the HCWD emits a discreet signal (vibration, sound, blink, etc.) to indicate to the HCW that a new patient cannot be touched without risk of a possible nosocomial contamination.

**Figure 4:**
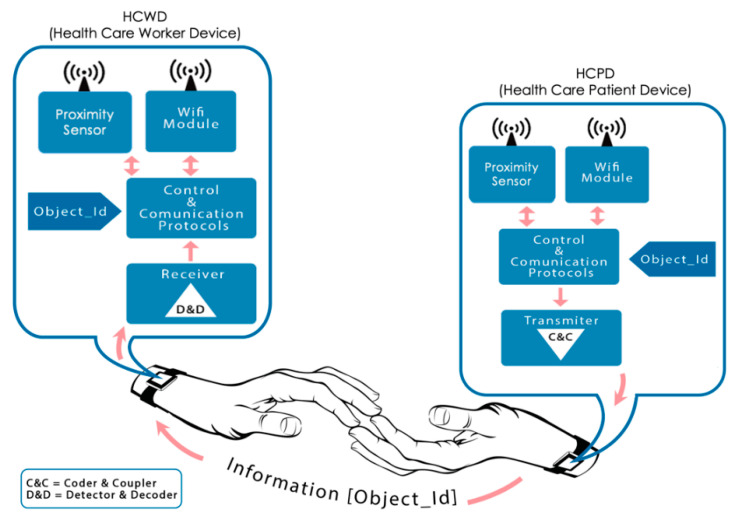
Touch detection system.

Figure 4 focuses on the proposed sensorization based on skin contact. This system is based on a communication device, implemented by means of a microcontroller and a new communication subsystem, which is able to transmit and receive information through the skin. A further development of this system would be implemented in a small bracelet worn by the involved agents. As it can be seen in Figure 4, a C&C (Coder & Coupler) and D&D (Detector & Decoder) have to be designed to allow this interbody communication. From our point of view, the standard IEEE802.15.6 is the most appropriate for this purpose. The next section details the implementation of both C&C and D&D, following this standard.

## 4. System Description

The aforementioned skin contact detection sensor is based in an implementation of the physical layer (HBC-PHY) of IEEE 802.15.6 standard [18], extending its application beyond a single body. In this way, our sensor detects the skin touch between humans through the transmission and reception of HBC compatible signals, according with the standard. It must be remarked that, for this application, the compatibility of data transmission signals with the standard is complete.

The viability of the sensor has been tested by means of a prototype, consisting of two devices. On one hand, a transmitter device emits a well-known identification code over the skin of one of the involved persons. On the other, a receiver device located in other person tries to detect the identification code. When the skin of both persons touches, the transmitted signal propagates from one body to the other, and it may be detected by the receiver device. 

Metallic electrodes have been used to couple both transmitter and receiver devices on human skin. These electrodes were selected due their excellent conductivity performance on the human body, achieving a better signal quality and thus enhancing the results. Later development phases would rely on metallic contacts, shown in bracelets in Figure 3 and Figure 4, assuming worse quality signals in the receiver device, but providing a higher commodity to the final users.

**Figure 5:**
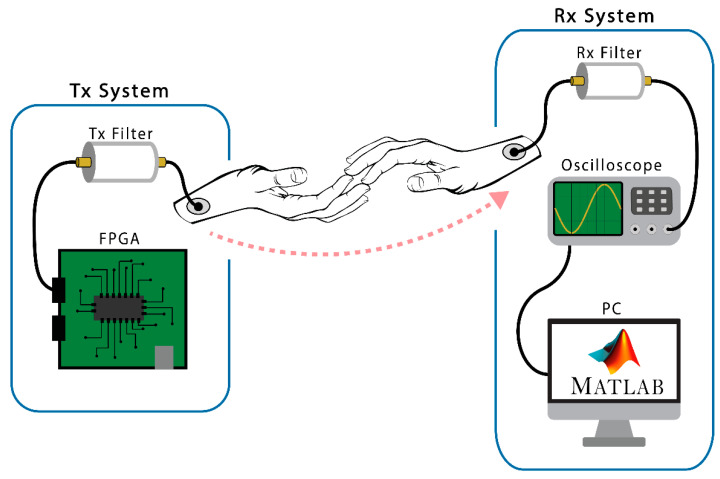
Scheme of the test prototype.

For this first stage in prototyping, a FPGA device has been selected to implement the transmitter device. A FPGA is capable of generating high-rate digital signals in an optimal way, also offering high reliability. The receiver device requires high power algorithms to reconstruct and understand the received signal. Thus, in this first version, a MATLAB environment on PC has been selected. The signals received from metallic electrodes are captured by means of an oscilloscope, allowing both signal observation and conditioning. This configuration is both powerful and flexible and facilitates the proving and evaluation of different receiving algorithms. Figure 5 shows a general schema of the prototype. The next paragraphs will describe the characteristics of both modules in a more detailed way. 

We have chosen to use this combination, since it is an optimal development environment in this prototyping phase, as it is possible to explore and debug different reception algorithms and carry out a multitude of tests and experiments in an agile and simple way. Our methodology follows a philosophy based on a top-down approach, starting with high abstraction development systems, and looking for maximum efficiency at each stage of the prototype. Later, in function of the results, we will choose the hardware elements of the final system according with these experiments. 

### 4.1. Transmitter Module

The transmitter module is made up of a Digilent © Nexys 4 DDR/Nexys A7 Artix-7 [46,47] FPGA development board. The necessary hardware has been designed on this board to generate the signals that will be transmitted through the body. Thus, each of the blocks proposed by the 802.15.6 standard to transmit IBC frames has been encoded in VHDL language. The synthesis of this code was programmed in the FPGA. As mentioned above, these signals are based on the specification of the standard [4], whose fundamental elements are a series of Gold and Walsh codes on which the different fields or blocks of an IBC frame are based.

The first block in the sequence is the PLCP preamble, which is made up of the repetition of four identical and consecutive Gold codes, whose generation method is based on a series of polynomials with a sequence of initial values established by the standard. The second block, called SFD/RI, is made up of another Gold code that is generated with the same polynomials, but different initial values, which results in a different Gold code. A Feedback Shift Register has been used to generate them. It is made up of several consecutive state memories where the binary sequences are stored and shifted through shift registers synchronized with clock signals.

**Figure 6:**
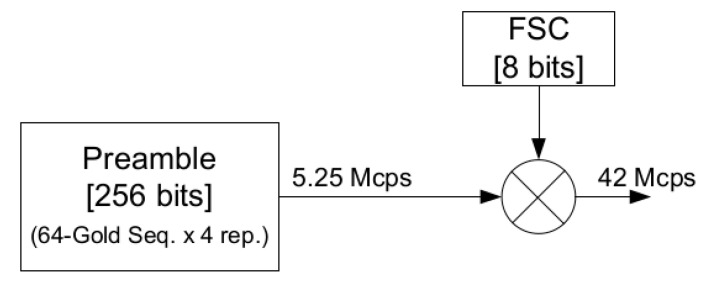
Preamble generation block diagram.

Once the 64-bit Gold sequences are generated, they are expanded with an 8-bit Frequency Shift Code (FSC) with a clock frequency of 42 MHz, resulting in a signal centered at 21 MHz, as shown in the Figure 6.

**Figure 7:**
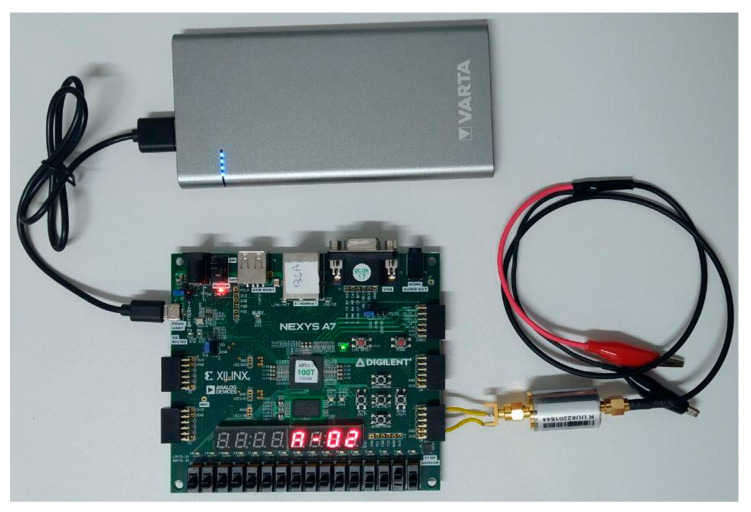
Experimental prototype: transmitter module.

Therefore, the VHDL entities in charge of generating the signals of the different Gold codes expanded with the FSC have been implemented. As seen in Figure 7, the switches and displays provided by the board have been used to make easier to change the code generated during the experimentation phase. Furthermore, the low consumption properties of the device make possible to supply it with batteries, which provides the isolate from power network of the transmitter and receiver systems lightly and efficiently.

**Figure 8:**
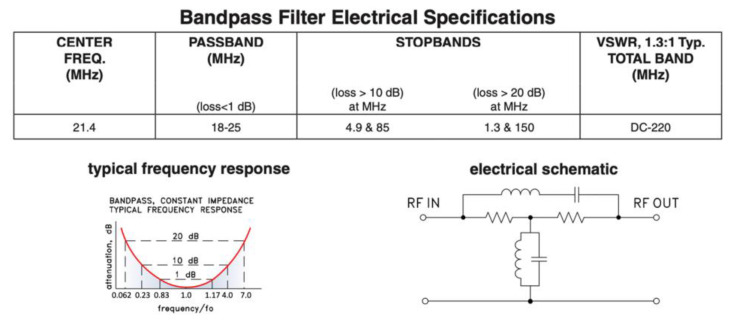
Filtering stage and frequency response.

Finally, the generated signal passes through an output filter, before inducing it through the body through common electrocardiogram electrodes attached to the body. This filtering is responsible for eliminating harmonics that may be harmful to humans or interfere with medical instruments (400 MHz band). For this reason, a passive band-pass filter of Mini-Circuits SIF-21.4+ [48] has been chosen. Its characteristics, shown in Figure 8, satisfy the spectral mask recommended by the standard (Figure 2).

### 4.2. Receiver Module

The receiver module comprises a connected electrode to the skin, from which signals are collected. After that, the input filter eliminates the noise at 50 Hz produced by the galvanic coupling with the electrical network. It also rejects any signals out of the FSDT band, and any glitch caused by the electrode amplifier. This filter has the same characteristics than the output filter discussed in the previous section (see Figure 8).

An Agilent Technologies DSO6014 [49] oscilloscope is used to capture the data after filtering the incoming signal. This device offers a 100 MHz bandwidth and 2 Gigasamples per second. It is used with the objective of demonstrating the feasibility of our proposal, and prior to selecting specific hardware, may be substituted by any ADC (Analog to Digital Converter) device with similar features.

The oscilloscope transmits the captured data to a PC provided with the MATLAB software [50], to carry out the processing. 

**Figure 9:**
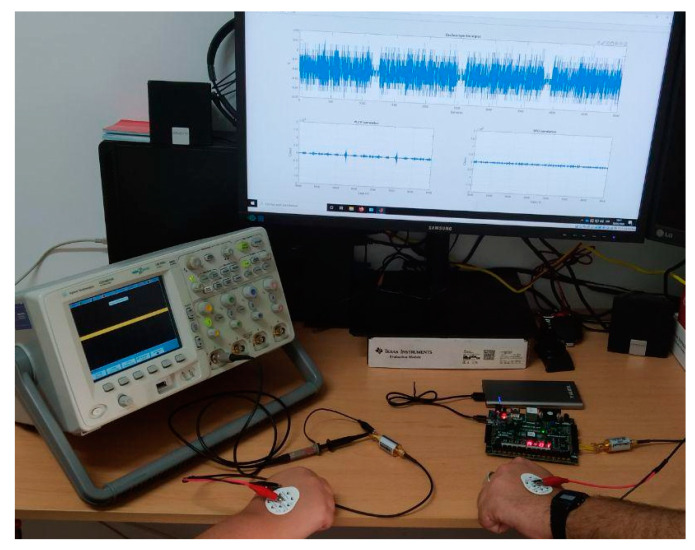
Experimental prototype.

The Gold codes, as a pseudo-random sequence, were designed to offer good orthogonality and correlation characteristics, which enhance the performance of correlation algorithms used to detect and identify the different codes. The MATLAB environment has been very useful when implementing these algorithms and generating the graphs and other forms of representation that show the results. Figure 9 shows the complete prototype used. In this prototype, the receiver module is permanently in listening mode, waiting for a valid signal. The receiver remains permanently capturing the signal present in the medium and applying the correlation algorithms to identify any of the different Gold codes. The algorithm generates a continuous signal with the correlation index and the Gold code. When this signal exceeds the established threshold, the corresponding Gold code (Object ID) is detected. As stated in Section 3, this contact will be communicated through WiFi to the Control Center. From this situation, if the HCW gets near of other patient before a cleaning action, the proximity sensor will detect this approach in “unsafe” mode, a warning event will be generated (vibration, sound, or blink in the HCWD) and the Control Center will be informed of this protocol violation.

It should be noted that all the previously described functions will be implemented in near future in a small wearable bracelet. A dedicated PCB (Printed Circuit Board) will be designed. Thus, the capture functions accomplished by the oscilloscope will be in charge of an ADC. The IEEE 802.15.6 protocol will be implemented in a small FPGA for both transmission and reception. For the latter, a microcontroller or DSP (Digital Signal Processor) will execute the algorithms (that are now implemented in the MATLAB tool) to process the incoming reception signal. The microcontroller will also control the WLAN communication controllers and the application.

## 5. Experimental

A series of experiments have been carried out to demonstrate the viability of the proposal, validate the design and demonstrate the feasibility of the interbody communication. The success of these experiments proved that our prototype can detect and identify contact between two humans. Likewise, when their identification codes are transmitted between them through the skin, it is possible to identify these persons.

Our experiments involve two people. One of them, the sender (HCP), is connected through a metallic electrode with the transmitter system: the FGPA with a filtering stage and battery powered. The other, the receiver (HCW), also uses a metallic electrode to be connected with the oscilloscope, which works like an analog to digital converter. The oscilloscope output signal is provided to the MATLAB programming environment to be processed, as shown in Figure 9. 

As capacitive coupling is used, just one electrode per person is required. Shielded cables were also used to avoid the antenna effect in the cable and ensure that the communication is carried out through the skin. To avoid common grounding through cables and appliances, the emitter module’s FPGA board is powered by an external battery. In this way, the common ground of the power lines will not generate a signal return path between the transmitter and the receiver.

In this experiment, the sender simulates to be a HCP, provided with a HCPD, and is constantly transmitting the Gold code of an IBC frame. The receiver, that simulates to be an HCW wearing a HCWD, is waiting for the reception of an IBC frame.

The Object_ID transmitted from the HCPD is variable. It may be selected with the switches included in the FPGA development board. This makes possible the simulation of the contact between different patients, verifying the detection of each one of the codes and displaying the data through the PC monitor. Three stages of this experiment must be identified by the HCWD:

1. HCPD is transmitting any Gold code, but there is no skin contact between the subjects.
2. When subjects are in contact, the HCPD is transmitting the Gold code corresponding to the PLCP preamble block (Object_ID_1).
3. When subjects are in contact, the HCPD is transmitting the Gold code corresponding to the SFD/RI block (Object_ID_2).

## 6. Results and Discussion

**Figure 10:**
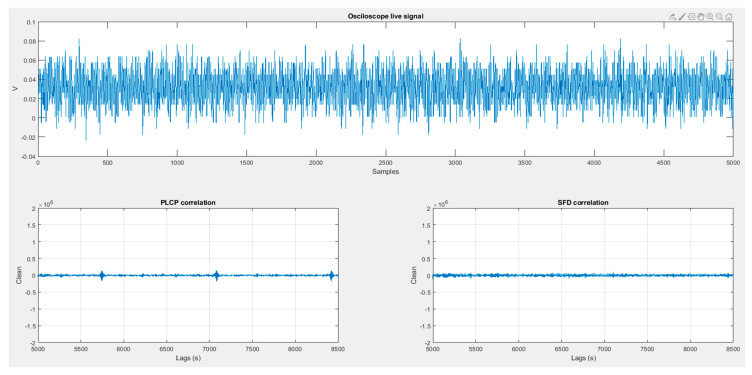
No contact situation.

**Figure 11:**
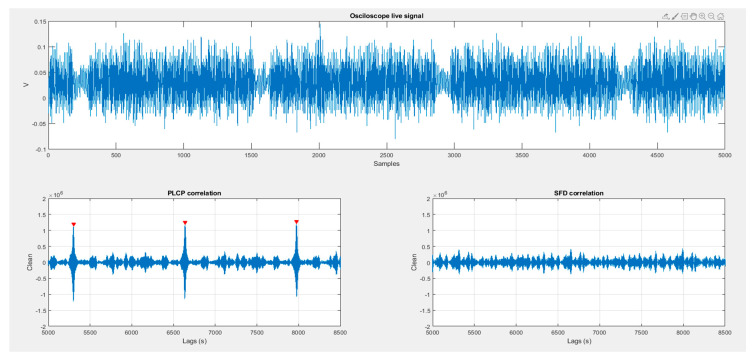
HPC and HPW contact when PLCP id-code is being transmitted.

**Figure 12:**
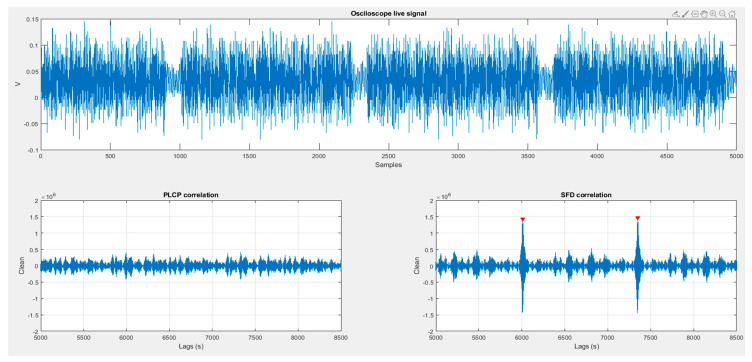
HPC and HPW contact when SFD ID-code is being transmitted.

This section presents the results of the aforementioned experiments. Figure 10 shows a non-contact situation. Figure 11 and Figure 12 reflect the results of the correlation with different Gold Codes in a contact situation.

In all Figure 10, Figure 11 and Figure 12, the received signal, captured by the oscilloscope, is shown in the upper panel. Lower panels show the results of correlating the received signal with both preselected Gold codes (PLCP Gold code and SFD/RI Gold code) which are also used as Object_ID_1 and Object_ID_2. 

The results of the different experiments are commented in more detail below:

### 6.1. Stage 1

In this first stage (Figure 10) the HCPD continuously transmits the PLCP Gold code. The hands of both HCP and HCW are quite close (about 1 cm) but there is not skin contact. It may be observed that the signal causes a small induction on receiver (about half of a contact signal), but as observed in the PLCP correlation panel, the correspondence is not enough to produce a false positive.

### 6.2. Stage 2

Figure 11 illustrates the results of this second part of the experiment. The HCPD transmits continuously the PLCP Gold code, and there is skin contact between HCP and HCW. The received signal is clearer than in the previous case, and the lower left panel shows many correlation matches (signaled red peaks) where the correlation with the right ID-code exceeds the predefined threshold. On the lower right panel, the correlation with the SFD keeps in a low index, far below the threshold that would cause a false positive.

### 6.3. Stage 3

Figure 12 shows a similar situation, where skin contact between HCP and HCW transmits a Gold code. But in this third stage the Object_ID being transmitted is the SFD/RI Gold code. As observed in Figure 11, received signal is clearer that the one shown in Figure 10, identifying that there are contact between the persons. However, the correlation with PLCP Gold code is negligible, whereas SFD Gold code correlation overcomes the detection threshold, as expected.

## 7. Conclusions and Future Work

In this paper, a new sensing environment has been presented. It is based on the use of Intra Body Communications (IBC) to transmit through the skin of a person an identifier that may be received by the receiver located in other person when a physical skin-to-skin contact exists. We have demonstrated through experimentation the feasibility of using the intra body communications standard for interbody communications. Many interesting advances have been required to provide this sensorization system. First of all, skin touch detection has been studied, and no sensor has been found that can identify the agents involved on the contact. As indicated in the Introduction, existing systems for the hand hygiene protocol are not able to detect physical contact between medical personnel and patients. Those systems only assume that the contact exists when two persons are close. Our proposed sensor ensures whether there has been contact or not. Therefore, it can be considered a clear advancement in this domain. On second place, the use of a communication environment based on IEEE 802.15.6 standard foresees that many applications may cooperate in an open environment towards a really smart sensing ecosystem. No commercial devices in these lines—touch identification and standard IEEE 802.15.6 HBC communications—are available, and only a very few research approaches have been documented. Beyond them, in this proposal a real implementation of both sensing environment and IBC communication devices have been fulfilled and experiments demonstrate its feasibility.

The proposed sensorization system may be considered as a complete novelty, as far as it solves in an efficient manner the detection of human contact. It has been applied to a state-of-the-art non-resolved problem with a huge impact on overall health of the society. Considering that about 20% of hospital patients get affected by nosocomial infections, this proposal may significantly reduce the incidence of these accidents. 

IBC communication and detection devices can also be considered a very interesting contribution. We are pioneering the making of a system that increases the application areas of the IEEE 802.15.6 HBC standard. This prototype may be considered an excellent workbench for future research in fields such as wearables, implants, or further developments using this standard. 

Regarding of the proposed application, this skin based sensorization technology can be also used in other applications, where information is required when a person touches an object. For instance, this could be useful in industrial applications (Smart Factory environment), food production and handling, education, games, and ludic applications, and so forth. 

Our next advances in these fields are focused on the miniaturization of the transmitter and receiver into a wearable bracelet, which will include IBC communications, skin electrode and WLAN communications. This bracelet could monitor the patients’ constants, too. The integration with the Cloud environment of a real hospital facility and the deployment in a real situation is being considered. The upload of these data to the hospital Control Centre would be very useful towards a real SmartHospital environment.
